# Electronic Videoendoscopy of Laryngeal Lesions Using a New Type of Rhinolarynx Endoscope Portion

**DOI:** 10.1155/DTE.4.199

**Published:** 1998

**Authors:** Masahiro Kawaida, Hiroyuki Fukuda, Naoyuki Kohno

**Affiliations:** 1 Department of Otolaryngology Tokyo Metropolitan Ohtsuka Hospital 2-8-1, Minamiohtsuka Toshima-ku Tokyo 170 Japan; 2 Department of Otolaryngology Keio University School of Medicine 35 Shinanomachi Shinjuku-ku Tokyo 160 Japan; 3 Department of Otolaryngology Juntendo University School of Medicine 2-1-1, Hongo Bunkyo-ku Tokyo 113 Japan

## Abstract

Patients with laryngeal lesions were observed and the lesions were recorded with an electronic videoendoscope system using the PENTAX EPM-3300 video processor and the PENTAX VNL-1330 endoscope portion. The electronic videoendoscope system differs from the conventional fiberoptic endoscope connected to a video camera in that a small monochrome charge-coupled device (CCD) chip is built in the tip of the endoscope portion. The PENTAX VNL-1330 rhinolarynx endoscope portion has a tip and insertion tube of approximately 4mm in outer diameter to allow its introduction through the nasal passages into the larynx. The dynamic color images provided by this system were superior to those obtained by a conventional rhinolarynx flexible fiberscope connected to a video camera in both quality and resolution of detail. This system should be useful in diagnosing laryngeal lesions.


*Diagnostic and Therapeutic Endoscopy*, Vol. 4, pp. 199–204
Reprints available directly from the publisher
Photocopying permitted by license only

© 1998 OPA (Overseas Publishers Association)
Amsterdam B.V. Published under license
under the Harwood Academic Publishers imprint,
part of The Gordon and Breach Publishing Group.
Printed in Singapore.

### Electronic Videoendoscopy of Laryngeal Lesions Using a New Type of Rhinolarynx Endoscope Portion

MASAHIRO KAWAIDA[a,*], HIROYUKI FUKUDA[b] and NAOYUKI KOHNO[c]

[a] *Department of Otolaryngology, Tokyo Metropolitan Ohtsuka Hospital, 2-8-1, Minamiohtsuka, Toshima-ku, Tokyo 170, Japan;*
[b] *Department of Otolaryngology, Keio University School of Medicine, 35 Shinanomachi, Shinjuku-ku, Tokyo 160, Japan;*
[c] *Department of Otolaryngology, Juntendo University School of Medicine, 2-1-1, Hongo, Bunkyo-ku, Tokyo 113, Japan*

*(Received 20 October 1997; Revised 20 November 1997; In final form 16 December 1997)*

**Patients with laryngeal lesions were observed and the lesions were recorded with an electronic videoendoscope system using the PENTAX EPM-3300 video processor and the PENTAX VNL-1330 endoscope portion. The electronic videoendoscope system differs from the conventional fiberoptic endoscope connected to a video camera in that a small monochrome charge-coupled device (CCD) chip is built in the tip of the endoscope portion. The PENTAX VNL-1330 rhinolarynx endoscope portion has a tip and insertion tube of approximately 4 mm in outer diameter to allow its introduction through the nasal passages into the larynx. The dynamic color images provided by this system were superior to those obtained by a conventional rhinolarynx flexible fiberscope connected to a video camera in both quality and resolution of detail. This system should be useful in diagnosing laryngeal lesions.**

*Keywords:* Electronic videoendoscope system, Rhinolarynx endoscope portion, Flexible fiberscope, Laryngeal lesions, CCD, Single-plate RGB sequencing method

### INTRODUCTION

An electronic videoendoscope system has been developed with a small charge-coupled device (CCD) chip as an ultra-miniature television camera built in the tip of the endoscope portion. This system enables to visualize laryngeal lesions with precision. The images obtained from the CCD chip are converted into electric signals and transmitted, and clear dynamic color images are reproduced on a color video monitor through a video processor. A new type of rhinolarynx endoscope portion has been developed with a small CCD chip built in the tip which is 4.1 mm in outer diameter. Observations of laryngeal lesions have been made with the conventional rhinolarynx flexible fiberscope connected to the video camera and electronic videoendoscope system using this new model of the endoscope portion. These images obtained are compared and discussed from the standpoint of diagnostic usefulness in this paper.

* Corresponding author. Tel.: +81-3-3941-3211. Fax: +81-3-3941-9557.

### EQUIPMENT AND METHODS

Electronic videoendoscopic examinations of the larynx were conducted by introducing the new rhinolarynx endoscope portion, the PENTAX VNL-1330 (Fig. 1(a)). The specifications of this endoscope portion are shown in Table I. This portion has a small monochrome CCD chip built in the tip, which functions as an ultra-miniature television camera in outer diameter of 4.1 mm. The insertion tube of this endoscope portion has an outer diameter of 4.2 mm (Fig. 1(b)). This endoscope portion is connected to a PENTAX EPM-3300 video processor that contains a xenon light source (Fig. 2). This system uses a single-plate red–green–blue (RGB) sequencing method which is described below.

(a)

(b)

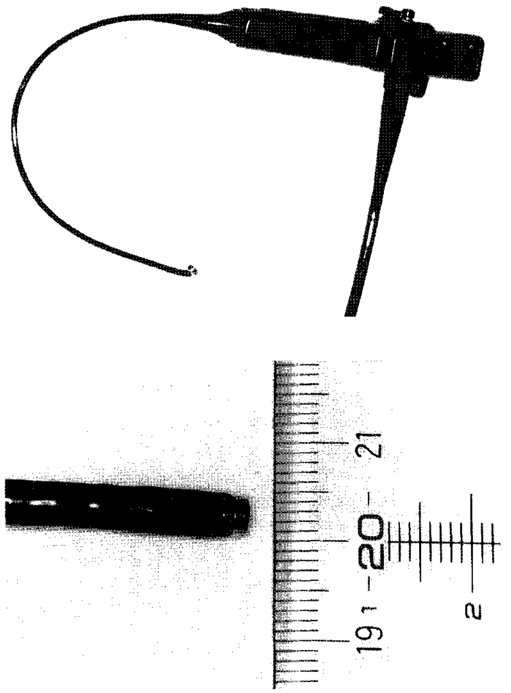

FIGURE 1 The PENTAX VNL-1330 rhinolarynx endoscope portion. (a): The external view of the PENTAX VNL-1330 endoscope portion. (b): The outer diameter of the tip of the endoscope is 4.1 mm.

TABLE I The PENTAX VNL-1330 rhinolarynx endoscope portion: specifications

| | | |
|---|---|---|
| Optical system | Field of view | 85° |
| | Direction of view | Forward viewing |
| | Depth of field | 3–50 mm |
| Bending section | Range of tip bending | Up 130°, Down 130° |
| Distal end | Outer diameter | 4.1 mm |
| Insertion tube | Outer diameter | 4.2 mm |
| Working length | | 300 mm |
| Total length | | 515 mm |
| Video processor | | PENTAX EPM-3300 |

White light obtained from the light source within the PENTAX EPM-3300 video processor sequentially illuminates the target through a rotating wheel filter that provides the three primary colors, red, green and blue. The RGB light components reflected from the target are sequentially detected by the monochrome CCD chip and are converted into electric signals. These signals are transmitted to the three image memories in the video processor. Dynamic color images are then reconstructed and projected onto the screen of the SONY PVM-1442Q color video monitor (Fig. 2).

Various recording devices, such as a SONY U-MATIC VO-7600 video tape recorder and a SONY UP-5000 color video printer, are also used in this system. A freeze-frame facility is provided to this system and a frozen frame can be printed with a color video printer. A subscreen mode permits simultaneous viewing of frozen and dynamic images through the main screen and sub-screen on the color video monitor. Patient information, such as name, age, sex and identity number, can also be simultaneously input onto the screen through a keyboard. The control mechanisms of the endoscope portion resemble those of conventional rhinolarynx flexible fiberscope.

When a patient is endoscopically examined using this system, surface anesthesia with 4% lidocaine hydrochloride spray is applied in the patient's nasal cavity. The patient is examined in the seated position. The insertion tube of the endoscope portion is then passed through the nasal passages and introduced into the larynx (Fig. 3).

In our study, the patient is examined with the system using a PENTAX FNL-10P2 rhinolarynx

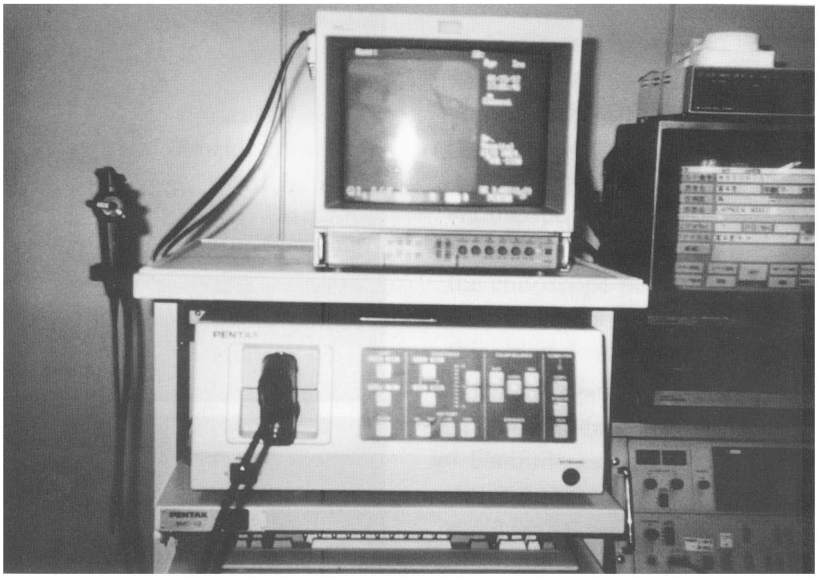

FIGURE 2 The PENTAX EPM-3300 video processor and SONY PVM-1442Q color video monitor.

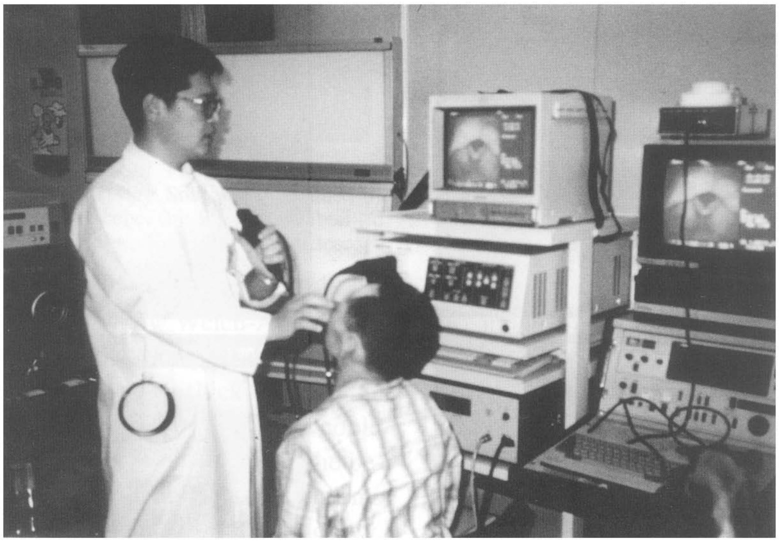

FIGURE 3 The performance of electronic videoendoscopic examination using the PENTAX VML-1330 rhinolarynx endoscope portion.

flexible fiberscope connected to the light source within the PENTAX EPM-3300 video processor and TOSHIBA IK-C30 CCD video camera. Electronic videoendoscopic examination followed in the same patient. The endoscopic findings on laryngeal lesions observed by both systems were recorded by the same video tape recorder, and still color images were printed by the same color video printer. The still images obtained by the system using the rhinolarynx flexible fiberscope connected to the video camera were compared with those obtained by the electronic videoendoscope system.

### SUBJECTS

Subjects in this study were a 66-year-old man with an anterior supraglottic hemangioma (Patient 1), a 72-year-old man with a laryngeal web (Patient 2) and a 43-year-old man with a nonspecific granuloma of the left vocal process (Patient 3).

### RESULTS

Still color images obtained by the system with a rhinolarynx flexible fiberscope are shown in Figs. 4(a), 5(a) and 6(a). Still color images obtained by freeze-frame facility with the electronic videoendoscope system are shown in Figs. 4(b), 5(b) and 6(b).

The quality and resolution of the images obtained with this electronic videoendoscope system were superior to those obtained with the flexible fiberscope in each case. Color reproductions with this electronic videoendoscope system appeared to be real. The maneuverability of the endoscope portion was as easy as that of a conventional rhinolarynx flexible fiberscope.

### DISCUSSION

An electronic videoendoscope system has been developed in which a small CCD chip is located at the tip of the endoscope portion as an ultraminiature television camera. The CCD chip was

(a)
(b)

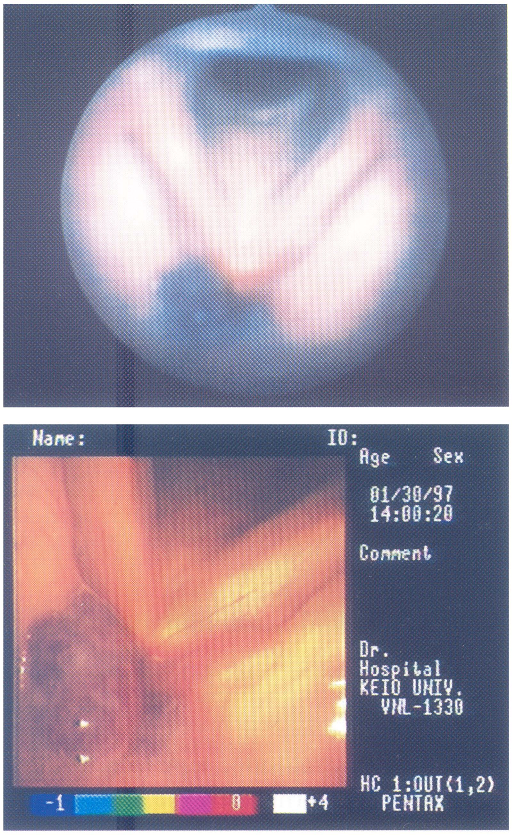

FIGURE 4 Laryngeal findings of Patient 1 (Anterior supraglottic hemangioma). (a): Still image obtained by the system with a flexible fiberscope. (b): Still image obtained by freeze-frame facility with the electronic videoendoscope system.

(a)
(b)

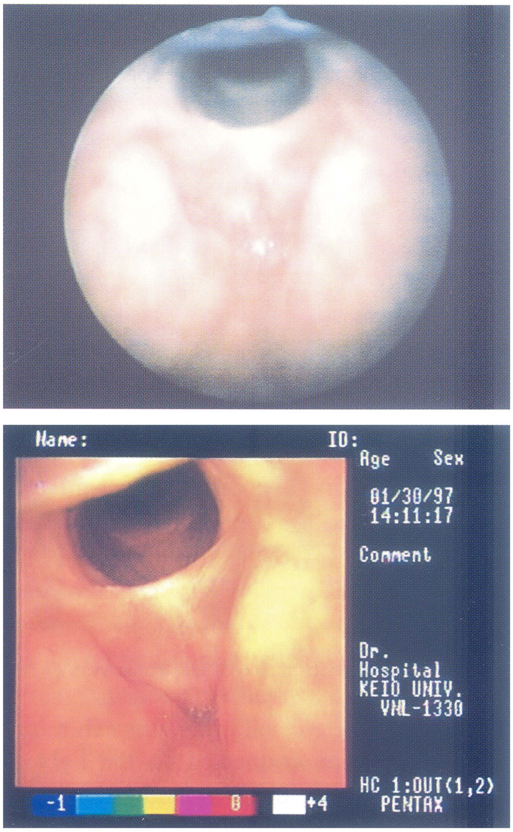

FIGURE 5 Laryngeal findings of Patient 2 (Laryngeal web). (a): Still image obtained by the system with a flexible fiberscope. (b): Still image obtained by freeze-frame facility with the electronic videoendoscope system.

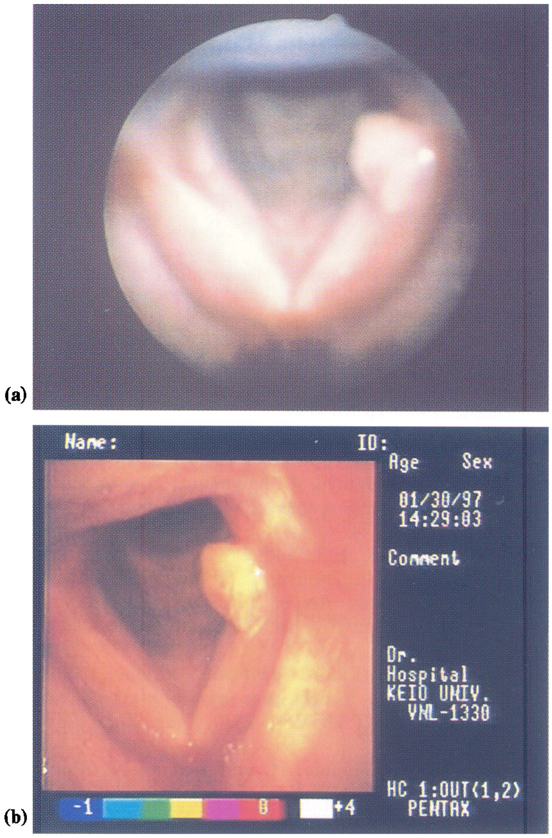

FIGURE 6 Laryngeal findings of Patient 3 (Nonspecific granuloma of left vocal process). (a): Still image obtained by the system with a flexible fiberscope. (b): Still image obtained by freeze-frame facility with the electronic videoendoscope system.

first utilized in endoscopy by Welch-Allyn, Inc. Tips and insertion tubes of early models of the endoscope portion were rather thick due to the large size of the CCD chip. These systems could only be used in the gastrointestinal tract in the beginning [1–4]. Electronic videoendoscope systems could not be easily applied in otolaryngology, because the rhinolarynx endoscope portion required a thinner outer diameter of the tip and insertion tube to pass through the patient's nasal passage. When a smaller CCD chip became available, the system could be used in the field of bronchology [5]. The PENTAX VNL-1530 rhinolarynx endoscope portion with an approximately 5 mm outer diameter tip and insertion tube was developed by Asahi Optical Co., Ltd. in 1993. In 1996, a similar model of a rhinolarynx endoscope portion was released by Olympus Optical Co., Ltd. The authors clinically performed endoscopic examinations of laryngeal lesions with these models [6,7]. Recently, Asahi Optical Co., Ltd. has developed the PENTAX VNL-1330 rhinolarynx endoscope portion which has a thinner outer diameter of the tip and insertion tube. The tip of the endoscope portion is 4.1 mm and the insertion tube is 4.2 mm in outer diameter. The system with this model was used in this study to observe laryngeal lesions.

The three basic components of an electronic videoendoscope system are an endoscope portion, a video processor containing a light source and a color video monitor. In this study, the PENTAX VNL-1330 rhinolarynx endoscope portion, PENTAX EPM-3300 video processor and SONY PVM-1442Q color video monitor were used with other optional accessories such as a video tape recorder and a color video printer. This system adopts the single-plate RGB sequencing method using a monochrome CCD chip. A monochrome CCD chip can only provide black and white signals. Because the CCD chip built in the tip of the endoscope portion is durable and shock-resistant, the electronic videoendoscope system is not fragile with handling. On the other hand, fiberoptic bundles which is built in the flexible fiberscope as an image-guide are prone to breakage with repeated uses. Endoscopic examination with the system using this endoscope portion could be easily performed through the patient's nasal passage into the larynx.

The main objective difference between the images from the electronic videoendoscope system and the images from the flexible fiberscope attached to a color video camera is the quality of the dynamic color images obtained. In the images obtained with flexible fiberscope attached to a color video camera, honeycomb pattern exists and an optical interference which is known as the "Moire effect" is occasionally seen on the screen of the color video monitor. The electronic videoendoscope system provides the examiners with clear and high qualitative color images which are transmitted in the form of electric signals and reproduced on the color video monitor. Therefore, the problem of obtaining a honeycomb pattern and "Moire effect" with the flexible fiberscope attached to a color video camera is avoided with this system. In this study, images obtained by the system using a flexible fiberscope and a video camera appeared rough with a honeycomb pattern. Images provided by the electronic videoendoscope system were superior to those obtained by the system using a flexible fiberscope and a color video camera with regard to resolution of details. The electronic videoendoscope system using the PENTAX VNL-1330 endoscope portion appeared to be extremely useful in the endoscopic diagnosis of laryngeal lesions.

There is one problem in the clinical use of the electronic videoendoscope system with a single-plate RGB sequencing method. Laryngostroboscopic observation is the most practical way of determining the vibratory mode of the vocal folds during phonation [8–10]. However, the laryngostroboscope cannot connect with the electronic videoendoscope system using a single-plate RGB sequencing method and a rotating wheel filter. In order to observe and record stroboscopic images with an electronic videoendoscope system, another method, such as one using a single-plate color CCD chip, must be necessary. The color CCD chips simplify color acquisition, but such chips are considerably larger than the monochrome CCD chips used in the present system. Consequently, the tip and the insertion tube of the endoscope portion have to be larger, and cannot be passed through the patient's nasal passage.

### *References*

[1] Sivak, M.V. and Fleisher, D.E. Colonoscopy with a video endoscope: Preliminary experience. *Gastrointest. Endosc.* 1984; **30**: 1–5.

[2] Classen, M. and Phillip, J. Electronic endoscopy of the gastrointestinal tract: Initial experience with a new type of endoscope that has no fiberoptic bundle for imaging. *Endosc.* 1984; **16**: 16–19.

[3] Matek, W., Lux, G., Riemann, J.F. *et al.* Initial experience with the electronic endoscope. *Endosc.* 1984; **16**: 20–21.

[4] Niwa, H., Kawaguchi, A., Miyahara, T. *et al.* Clinical use of new video-endoscopes (EVIS 100 and 200). *Endosc.* 1992; **24**: 222–224.

[5] Ono, R., Edell, E.S. and Ikeda, S. Newly developed bronchoscope. In: Inouye, T., Fukuda, H., Sato, T. and Hinohara, T., eds. *Recent Advances in Bronchoesophagology.* Amsterdam: Elsevier Science Publishers, B.V. 1990: 49–53.

[6] Kawaida, M., Fukuda, H. and Kohno, N. Clinical experience with a new type of rhino-larynx electronic endoscope PENTAX VNL-1530. *Diag. Ther. Endoscopy.* 1994; **1**: 57–62.

[7] Kawaida, M., Fukuda, H. and Kohno, N. Rhinolarynx electronic videoendoscope system. In: McCafferty, G., Coman, W. and Carroll, R., eds. *Sydney '97 XVI World Congress of Otorhinolaryngology Head and Neck Surgery.* Bologna: Monduzzi Editore. 1997;1689–1692.

[8] Saito, S., Fukuda, H., Kitahara, S. *et al.* Stroboscopic observation of vocal fold vibration with fiberoptics. *Folia. Phonoatr.* 1978; **30**: 241–244.

[9] Yoshida, Y., Hirano, M., Yoshida, T. *et al.* Strobofiberscopic colour video recording of vocal fold vibration. *J. Laryngol. Otol.* 1985; **99**: 795–800.

[10] Hirano, M., Yoshida, Y., Yoshida, T. *et al.* Strobofiberscopic video recording of vocal fold vibration. *Ann. Otol. Rhinol. Laryngol.* 1985; **94**: 588–590.

